# LOcating Non-Unique matched Tags (LONUT) to Improve the Detection of the Enriched Regions for ChIP-seq Data

**DOI:** 10.1371/journal.pone.0067788

**Published:** 2013-06-25

**Authors:** Rui Wang, Hang-Kai Hsu, Adam Blattler, Yisong Wang, Xun Lan, Yao Wang, Pei-Yin Hsu, Yu-Wei Leu, Tim H.-M. Huang, Peggy J. Farnham, Victor X. Jin

**Affiliations:** 1 Department of Chemistry, Lanzhou University, Lanzhou, China; 2 Department of Biomedical Informatics, The Ohio State University, Columbus, Ohio, United States of America; 3 Department of Molecular Medicine, Institute of Biotechnology, University of Texas Health Science Center, San Antonio, Texas, United States of America; 4 Department of Biochemistry and Molecular Biology, Norris Comprehensive Cancer Center, University of Southern California, Los Angeles, California, United States of America; 5 Genetic Graduate Group, University of California-Davis, Davis, California, United States of America; 6 Human Epigenomics Center, Department of Life Science, Institute of Molecular Biology and Institute of Biomedical Science, National Chung Cheng University, Chia-Yi, Taiwan; McGill University, Canada

## Abstract

One big limitation of computational tools for analyzing ChIP-seq data is that most of them ignore non-unique tags (NUTs) that match the human genome even though NUTs comprise up to 60% of all raw tags in ChIP-seq data. Effectively utilizing these NUTs would increase the sequencing depth and allow a more accurate detection of enriched binding sites, which in turn could lead to more precise and significant biological interpretations. In this study, we have developed a computational tool, LOcating Non-Unique matched Tags (LONUT), to improve the detection of enriched regions from ChIP-seq data. Our LONUT algorithm applies a linear and polynomial regression model to establish an empirical score (ES) formula by considering two influential factors, the distance of NUTs to peaks identified using uniquely matched tags (UMTs) and the enrichment score for those peaks resulting in each NUT being assigned to a unique location on the reference genome. The newly located tags from the set of NUTs are combined with the original UMTs to produce a final set of combined matched tags (CMTs). LONUT was tested on many different datasets representing three different characteristics of biological data types. The detected sites were validated using *de novo* motif discovery and ChIP-PCR. We demonstrate the specificity and accuracy of LONUT and show that our program not only improves the detection of binding sites for ChIP-seq, but also identifies additional binding sites.

## Introduction

Next-generation sequencing technologies have been widely used to address many biological and medical questions on a genome-wide scale. For example, ChIP-seq was one of the first high throughput techniques that utilized massively parallel sequencing platforms to interrogate in vivo protein-DNA interactions [1]–[4] and histone modifications [1], [5]–[7]. More recently, sequencing techniques have been coupled with mRNA samples to measure gene expression (RNA-seq) [8]; other sequencing technologies include Hi-C [9]–[10] and BS-seq [11].

Despite the large number of computational tools, such as MACS [12], QuEST [13], SISSRs [14] and many other peak identification programs [15]–[21] for ChIP-seq data, and Cufflinks [22], Scripture [23] and SpliceTrap [24] for RNA-seq data, that have been developed to analyze genomic datasets generated from sequencing-based technologies, limitations in data analysis still exist. One big limitation is that most of the existing tools ignore non-unique tags (NUTs) that match the genome under study and merely focus on unique matched tags (UMTs). However, NUTs comprise up to 60% of all raw tags [25]. Effectively utilizing these NUTs would increase the sequencing depth and allow a more accurate detection of enriched binding sites, which in turn may lead to more precise and significant biological insights.

A few recent studies [26]–[27] have investigated the effectiveness of multi-matched tags in detecting new binding sites and discuss the relationship of the NUTs with repetitive regions. However, there are very few analytical tools currently available in the field to address NUTs from ChIP-seq data. The only executable tool from Chung et al [26] outputs many possible genomic locations for each NUT with ranked E values which makes it hard to determine the exact location for each NUT. Therefore, we have developed a computational tool, LOcating Non-Unique matched Tags (LONUT), that utilizes both UMTs and NUTS to improve the detection of binding sites for ChIP-seq data. Our LONUT algorithm applies a linear and polynomial regression model to establish an empirical score (ES) formula by considering two factors, the distance of NUTs to peaks identified using UMTs and the enrichment score for the peaks. Each NUT is assigned to one unique location on the reference genome based on the highest ranked ES. Then, the set of newly located tags from the NUTs is combined with the set of original UMTs to produce a final set of combined matched tags (CMTs). We tested the LONUT program on many different datasets in three study cases, representing three different types of data. In the first study (Study Case 1), all six ChIP-seq datasets from MCF7 cells, are publically available [28]. In the second study (Study Case 2), we used LONUT to analyze ChIP-seq data for a macromolecular complex in K562 cells comprised of a master regulator, a repressive histone modification mark and a histone methyltransferase. In the third study (Study Case 3), we used LONUT to analyze publically available ChIP-seq datasets for three transcription factors (TFs) in four cell types. The detected binding sites were validated using de novo motif discovery and ChIP-PCR. We show that our LONUT can not only improve the identification of binding sites for ChIP-seq data that are identified using only UMTs, but can also identify additional sites; thus, we demonstrate the specificity and accuracy of our tool LONUT.

## Results

### Overview of LONUT algorithm

**Figure 1:**
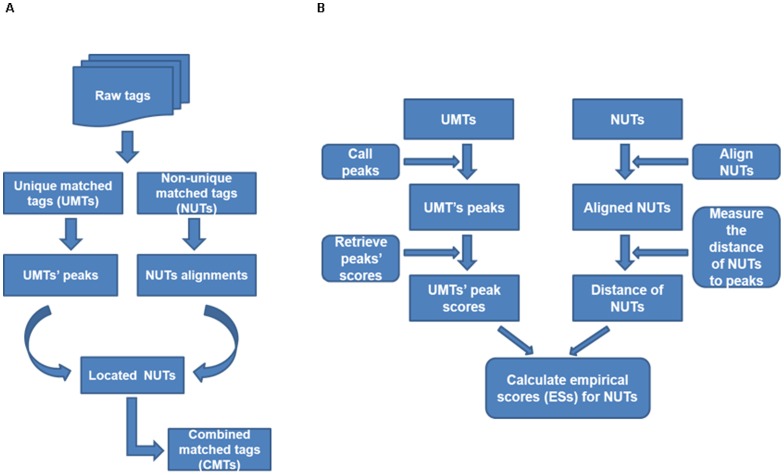
An overview of the LONUT algorithm. **A.** A flow chart summarizing the steps beginning with an initial set of raw tags to a final set of combined matched tags (CMTs). **B.** A graphic flow showing the detailed algorithm of locating each of NUTs to a unique reference genomic region with an ES formula derived from a linear and polynomial regression model.

The algorithm of LONUT is composed of two major steps (**Figure 1A**). In the pre-processing step, we obtain a set of all possible alignments for each raw tag in the dataset by utilizing a short-tag alignment tool, Bowtie aligner. The first step of LONUT is to divide the input dataset into two subsets: a set of UMTs and a set of NUTs based on the output dataset from the Bowtie aligned tags file. For the set of UMTs, we apply BELT [29], a tool developed in our laboratory to detect ChIP-seq binding sites, to identify the peaks (enriched binding regions) that will be used as a reference to assign the NUTs. We then establish an empirical score formula by considering two possible factors, the distance of each NUT to a peak (*d*) identified by BELT from UMTs and the enrichment score for the peak (*s*) (**Figure 1B**; also see next section). Then for each NUT having less than 100 possible genomic locations, we calculate an empirical score (ES) based on the empirical score formula. Based on the rank of the ES for all copies of the aligned tags for each NUT, the copy with the highest ES will be selected and each NUT will be assigned to one unique location on the reference genome. We then combine the set of newly located tags from the NUTs with the set of original UMTs to produce a final set of CMTs.

### Determination of ES formula

**Figure 2:**
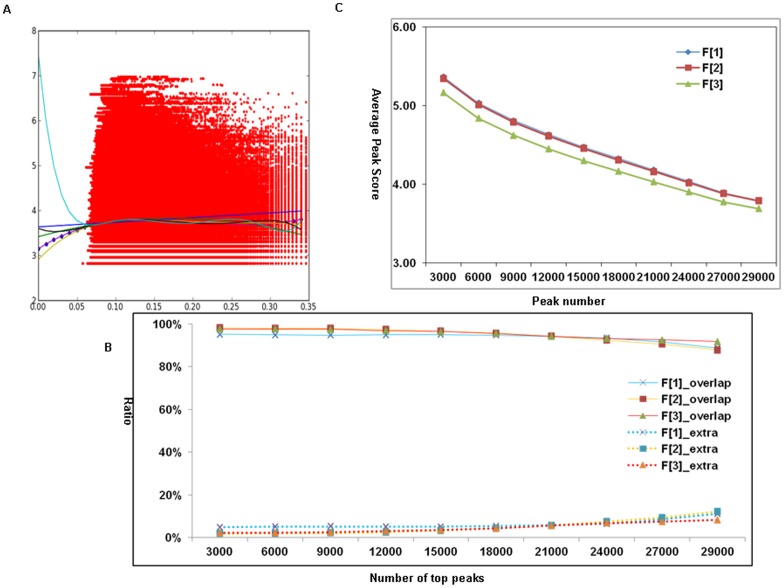
The determination of an ES formula using ChIP-seq data of H3K4me2 in MCF7 cell. **A.** A polynomial regression model to fit two vectors of data points of two influence factors (*d* and *s*) varying from degree 1 to 7 and we found that the data points fit the best with a degree one. X-axis is ln(*d*) for each NUT and Y-axis is *s* for the peak score for that NUT. **B.** The ratio of Overlap and Extra peaks with three ES formulas tested at different level of top peaks, showing a better performance for both ES F[1] and ES F[2] at all level of top peaks examined. **C.** An average peak score of CMT peaks with three ES formulas tested at different level of top peaks, showing that although ES F[2] maintains a higher average CMT peak score compared with ES F[1], its average Extra peak score is lower than that of ES F[1], illustrating that the Extra peaks derived from ES F[2] are much weaker that those from ES F[1].

To integrate two different influence factors (*d* and *s*) into an empirical score formula, we normalize them to the same numerical scale in order to equalize the impact of the two factors. We used a ChIP-seq dataset of H3K4me2 from MCF7 cells to derive the formula. The data is composed of a total of 2,910,475 raw tags, of which 2,417,878 (83%) are UMTs and 492,597 (17%) are NUTs. We found the ln scale of *d* is within the same scale of *s* after multiplying a normalized factor. We then applied a linear and polynomial regression model to fit two vectors of data points of two factors varying from degree 1 to 7 (**Figure 2A**). We found that the data points fit the best with a degree one. Thus, we derived the ES formula ES F[1] as shown below. To determine if the derived formula is the optimal one, we compared it to two other formula ES F[2] and ES F[3], in which only one of the influence factors is considered in each formula.

ES F[1]  = 1/ln(*d*)+0.0608*s.
ES F[2]  = 1/ln(*d*).
ES F[3]  = 0.0608*s.

Two aspects were considered to measure the efficiency of these formulas: 1) the similarity of UMT peaks and CMT peaks, i.e. the ratio of Overlap Peaks (the number of common peaks between UMT peaks and CMT peaks divided by the CMT peaks) and the ratio of Extra Peaks (the number of new peaks only in CMT peaks divided by CMT peaks); 2) the influence of LONUT on the density of peaks, i.e. the average peak scores of the Overlap and Extra peaks. Since the ratio of Overlap peaks represents the similarity between CMTs and UMTs, the higher ratio may indicate the quality of re-located NUTs is comparable to the original UMTs; while the higher ratio of Extra peaks indicates that LONUT has recovered a significant amount of NUTs and suggests a higher efficiency of the formula. We performed a comparison for all peaks (**Table S1 in File S2**) and subsets of different levels of top peaks (**Figure 2B**) to evaluate the efficiency for the three formulas in terms of the ratio of Overlap and Extra peaks of H3K4me2 data in MCF7 cells. The results showed a better performance for both ES F[1] and ES F[2] regardless of how many peaks were examined. The other aspect for measuring the efficiency of LONUT is to test the influence of LONUT on the density of CMTs and UMTs, which could reflect how LONUT changes the distribution of tags. We compared the average peak scores of UMT peaks, CMT peaks, Overlap peaks and Extra peaks on three formulas for all peaks (**Table S2 in File S2**) as well as subsets of different level of top peaks (**Figure 2C**), showing that although ES F[2] maintains a higher average CMT peak score compared with ES F[1], its average Extra peak score is lower than that of ES F[1], illustrating that the Extra peaks derived from ES F[2] are much weaker that those from ES F[1]. Since LONUT focuses on the extra tags in addition to UMTs, which are shown in Extra peaks, ES F[1] is better than ES F[2]. Since both the average CMT peak score and average Extra peak score of the results of ES F[3] are lower than those ES F[1], it is clear that ES F[1] has stronger and better performance. Taken together, our results demonstrated that ES F[1] is the best method for LONUT.

### Study case 1

**Figure 3:**
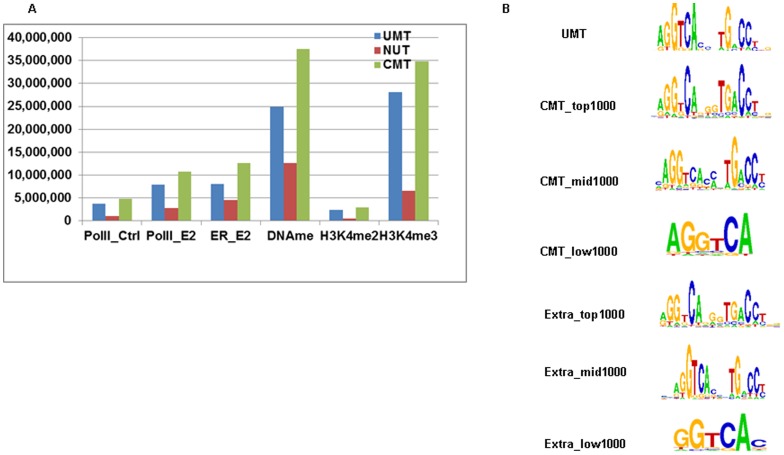
Study case 1: an evaluation of the performance of LONUT on six ChIP-seq datasets from MCF7 cells. **A.** A summary of tag distribution of UMTs, NUTs and CMTs. **B.** *De novo* motif discovery on ER_E2 ChIP-seq data using the ChIPMotifs, including motifs identified from the UMT peaks, Top, Middle and Lower 1000 CMT peaks, and Top, Middle and Lower Extra peaks.

In the first study case, we chose six datasets, including Estrogen Receptor (ER), RNA Polymerase II (Pol-II), histone modifications (H3K4me2 and H3K4me3), and DNA methylation (DNAme), all from the MCF7 cell line. The NUT ratio (measuring against all total tags in each dataset) ranged from 17% for H3K4me2 to 34% for DNAme with an average NUT ratio of ∼25% (**Figure 3A** and **Table S3 in File S2**). To demonstrate the efficiency of LONUT, we compared the peaks before running LONUT (peaks identified from UMT sets) and after running LONUT (peaks identified from CMT sets). We used our BELT program to call peaks at the same threshold and bin size for each pair of sets (UMT and CMT for the same data) with a FDR less than 10% for all datasets (see a summary of parameters used for the BELT in **Table S4 in File S2**). Interestingly, we found that the two active histone marks (H3K4me2 and H3K4me3) had the highest Overlap peaks and ratios (∼80%) and fewer Extra peaks were identified using LONUT (∼25%); for Pol-II, ER and DNAme, we found that the Overlap ratio was ∼40–60% (**Table S5 in File S2**). To understand what extent the NUTs contribute to the CMT peaks, we calculated the ratio of UMTs/CMTs ( = UMTs+NUTs) for each peak for CMT peaks set for ER and Pol-II data respectively. We first ranked CMT peaks based on BELT enriched peak score, and calculated the ratio of UMTs/CMTs by averaging every 100 peaks (**Figure S1 in File S1**), showing the ratio is decreasing along the decrease of the order of ranking peaks, but stable at 0.6 for Pol-II data and 0.35 for ER data. In addition, we compared the average of the peaks scores between the sets of UMT and CMT peaks (**Table S6 in File S2**), and found that the average of the CMT Peak Score is higher than the average of the UMT Peak Score, but lower than the average of the Overlap Peak Score, indicating that assigned NUTs are able to contribute to the strength of sites identified in ChIP-seq or DNAme datasets. We also observed that the average of the Extra Peak Score is lower than the average of CMT Peak Score and the average of Overlap Peak Score, but higher than the average of UMT Peak Score except for the two active histone marks data, illustrating that LONUT can identify enriched regions which are denser than the UMT peaks. Taken together, our results suggest that the newly identified peaks by LONUT are just as good as the original peaks identified by UMTs.

To test the accuracy of our LONUT algorithm, we applied ChIPMotifs [30] for *de novo* motif discovery using ChIP-seq peaks from ER_E2 data (**Figure 3B** and **Table S7**–**12 in File S2**). We used seven sets of peaks, one set was from the UMT Peak set, where we retrieved the Top 1000 peaks with a length of 300 bp for each peak extended 150 bp each side from the mid-point of the peak; r three sets were from the CMT Peak set and included a set of Top 1000 peaks, a set of Middle 1000 peaks and a set of Bottom 1000 peaks; the remaining three sets were from the Extra Peak set including a set of Top 1000 peaks, a set of Middle 1000 peaks and a set of Bottom 1000 peaks. We identified the canonical ER binding motif in all seven r sets, except that only half of ER motif was found in the sets of CMT and Extra Bottom 1000 peaks, demonstrating that the NUTs are indeed located to an accurate genomic location by our LONUT program.

### Study case 2

**Figure 4:**
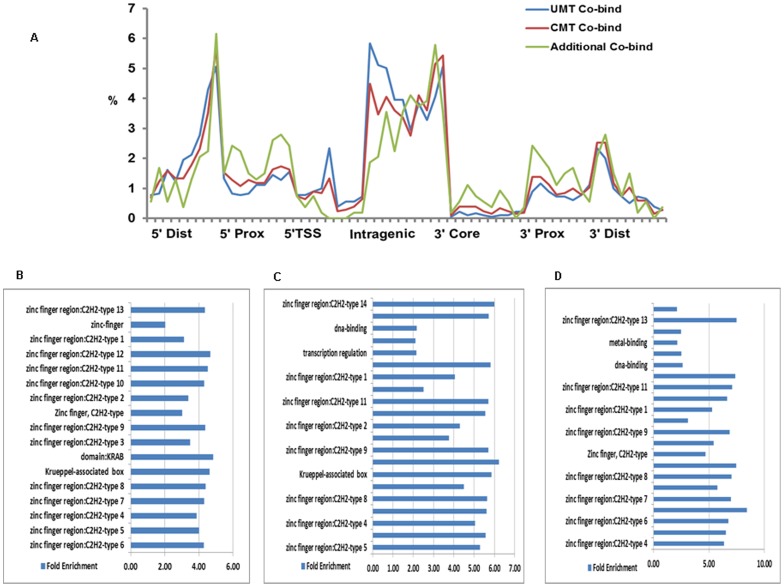
Genomic sites bound KAP1, SETDB1 and H3K9me3 identified in the UMT and CMT peaks sets. **A.** Location analysis showing the same trends of binding site distribution for all three co-bound sites using 2035 UMT, 2305 CMT and 682 additional peaks. **B**–**D.** GO analyses identified 757 genes in the common CMT peak set (**B**); 1047 genes in the common UMT peak set (**C**) and 274 genes in the additional 682 peak set (**D**); Zinc Finger C2H2-type genes are enriched in all three peak sets.

To further evaluate the effectiveness of LONUT on a macromolecular complex, we tested it on three ChIP-seq datasets, KAP1, SETDB1, and H3K9me3, from K562 cells. Our previous studies have shown that KAP1 and SETDB1 co-localize at sites of H3K9me3 [31], [32]. An overview of the tag distribution of UMTs, NUTs and CMTs in KAP1, SETDB1 and H3K9me3 in K562 cell (**Table S13 in File S2**) showed that NUT ratios for the three datasets were 26–34%. Similar to the first study case, we also performed a comparison of Overlap and Extra peaks for the UMT and CMT data in this study case (**Table S14**–**15 in File S2**). The results showed that more than 50% of the CMT peaks overlap with the UMT peaks for all three data, indicating that there exists a strong correlation between the UMT peaks and the CMT peaks. We next determined the sites in the genome that are identified as being bound by all three marks (the modified histone, the histone methyltransferase, and the KAP1 scaffold protein) using the UMT and the CMT peak sets. We found 682 new sites associated with 274 genes in the genome having all three marks when using the CMT peak sets. However, 201 of 274 genes were already in the list of 1,047 genes in the UMT peak sets (**Figure S2 in File S1**), indicating the newly identified genes in study case II are really new. Location analysis showed the same trends of binding site distribution for all three co-bound sets using UMT, CMT and additional peaks (**Figure 4A**). GO analysis also showed that the 274 genes corresponding to the additional 682 sites have the same functional categories of Zinc Finger C2H2-type genes as do the genes identified by the co-bound sites in the UMT and CMT peak sets (**Figure 4B**–**D**). These analyses demonstrate that LONUT is able to identify more commonly bound target loci for this complex.

### Study case 3

**Figure 5:**
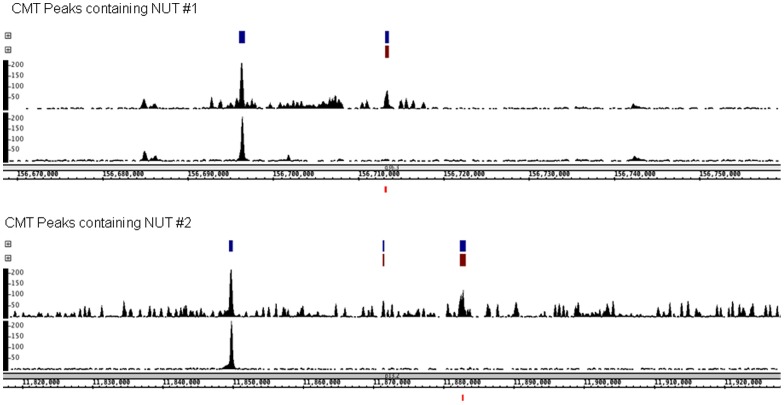
Screenshots of NUT regions tested by ChIP-PCR. The Integrated Genome Browser (http://bioviz.org/igb/) was used to visualize TCF7L2 ChIP-seq data in HCT116. The snapshots show called peaks for the CMT dataset (blue) and NUT peaks (red) above the raw read visualization tracks. In each panel, the top visualization track contains CMT tags and includes NUTs. The bottom visualization track contains only UMTs. Below the genomic coordinates for each snapshot, the small red box indicates the region for which PCR primers list in **Figure S3 in File S1**.

To test the performance of LONUT in identifying peaks from additional site-specific TF ChIP-seq datasets, in the third study case we chose publically available ChIP-seq data for three TFs, NRSF, TCF7L2 and YY1, from four different cell lines. We found that the overall NUT ratio for ChIP-seq of TFs was 30–40% (**Table S16 in File S2**). A comparison of Overlap and Extra peaks for the UMT and CMT data in this study case (**Table S17**–**19 in File S2**) showed that in most cases more than 40% of the CMT peaks overlap with the UMT peaks, indicating that there exists a strong correlation between the UMT peaks and the CMT peaks. However, ∼40–60% of the peaks in the 0.95 threshold CMT sets were newly identified due to relocation of NUTs. The comparison of the average peak scores for different sets of peaks including the UMT, CMT, Overlap and Extra peaks showed that the highest one is for the Overlap peaks, while the average peak scores of the CMT and Extra peaks for all four datasets are higher than the average peak score of UMT peaks, illustrating that adding the NUTs to UMTs reinforces the strength of previously identified peaks and creates new enriched regions of tags which are denser than the UMT peaks. An example of the tag densities in two regions of the genome in the UMT and CMT TCF7L2 datasets is shown in **Figure 5**. We also conducted ChIP-PCR on several 7 TCF7L2 NUT peaks (**Figures S3 in File S1**), showing that some of the NUT peaks were as strongly enriched as the previously identified TCF7L2 binding sites.

To further understand to the extent of the relationship of UMT and CMT TCF7L2 peaks, we examined a set of 7,800 very high threshold level UMT peaks for TCF7L2 (0.99 and bin-size of 150 bp). We found that 5,389 (69%) are overlapping with CMT peaks at the same level. However, all of the 7,800 peaks are overlapping with CMT peaks called at lower thresholds (**Table S20 in File S2**). This analysis indicates that while the majority of UMT peaks are retained in the CMT peak set, the signal intensities for some of newly formed CMT peaks are stronger than some of the original UMT peaks, thus resulting in some original UMT peaks being pushed lower in the ranked list and falling out of the top-ranked list of CMT peaks. In other words, the peaks are not “lost”, they are simply pushed into a lower ranking. An example of this is shown in **Figure S4 in File S1**.

**Figure 6:**
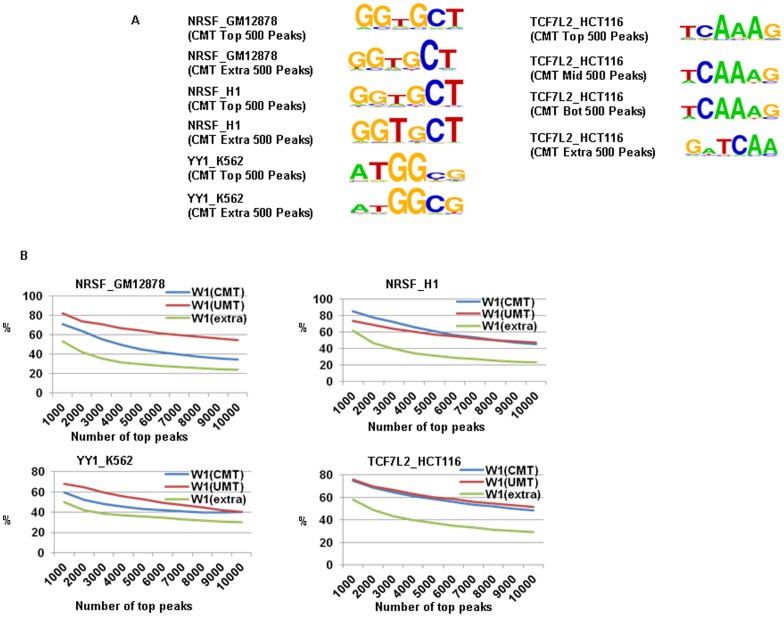
An evaluation of the performance of LONUT on ChIP-seq datasets of three TFs from four cell lines. **A.** *De novo* motif discovery on three TFs on four cell lines using the ChIPMotifs identifying the canonical binding motifs for all four data. For NRSF in GM12878 and H1 cells, and YY1 in K562 cells, we identified each canonical motif for both Top 500 CMT peaks and Top 500 Extra peaks. For the TCF7L2 data, we particularly examined it three subsets including top 1/3 peaks, middle 1/3 peaks and bottom 1/3 peaks. We were able to recover a 6-mer TCF7L2 core motif from all sets of peaks. **B.** A motif recovery plot showing that these 6-mer core motifs (W1) identified by our ChIPMotifs are present in a large percentage of the binding peaks with a gradually percentage reduction along more peaks consideration.

We also tested the accuracy of LONUT using *de novo* motif ChIPMotifs. We were able to identify the canonical binding motifs for all three TFs for both UMT and CMT peaks (**Figure 6A** and **Table S21**–**28 in File S2**). For the TCF7L2 data, we split the CMT peaks into three subsets including top 1/3 peaks, middle 1/3 peaks and bottom 1/3 peaks. We then selected 500 peaks from each subset and 500 peaks that were identified using CMTs but not identified using UMTs for the *de novo* motif analysis. We were able to recover a 6-mer TCF7L2 core motif from all sets of peaks. This supports that the newly formed peaks from CMTs are indeed true binding sites. We further analyzed recovery rates for identified binding motifs in both UMT and CMT peaks sets (**Figure 6B**), showing that these known motifs for each factor are present in a large percentage of the binding peaks with a gradually reduced percentage as more peaks are considered. For example, more than 80% of the top 1,000 peaks in each dataset from each cell type contain the 6-mer core motif (W1), with the percentage gradually dropping to ∼20% of all peaks. Interestingly, although many of the Extra peaks did contain the expected motif, smaller recovery rates were observed for Extra peaks for all four datasets.

Finally, we compared LONUT to another tool, developed by Chung et al [26], where they derived an iterative weighting scheme that also takes into account the number of tags mapped in the vicinity of that tag. We selected a best E value for each NUT from several E values output to combine to original UMTs since their tool outputs several possible different genomic locations for each NUT. We then used the same levels of thresholds for BELT peaks that we used to compare CMTs identified by LONUT and Combined Reads (CRs) identified by Chung's tool (**Table S29 in File S2**). We found that the overlap CMT peaks numbers between the two programs are very high (63%) at a relatively lower threshold of 0.95 with a bin size of 150 bp, and reaches 82% at a higher threshold of 0.99. The overlapping Extra peaks between two programs are 42.5% for the threshold of 0.95 with a bin size of 150 bp. This comparison showed that both tools tend to detect real peaks at higher level and LONUT has a comparable efficiency and accuracy to Chung's tool. Moreover, LONUT has a faster running speed and is easier to use (**Notes in File S2**).

## Discussion

An important challenge for computational biologists is to develop new software tools to analyze the large and increasingly varied amount of sequencing-based ‘omics data that is being generated by bench scientists. A big limitation of many existing tools in the field is that they do not consider non-unique matched tags (NUTs), creating a problem in the analysis of datasets from technologies such as ChIP-seq. To fill this gap, we have developed a program called LONUT (Locating Non-unique Matched Tags). LONUT improves the detection of the enriched regions for both ChIP-seq and MBD-seq data. Importantly, the ES formula, a core part of the LONUT, was derived using real datasets by a statistical regression model and thus is able to capture the underlying characteristics of biological meaning based on individual data types.

**Figure 7:**
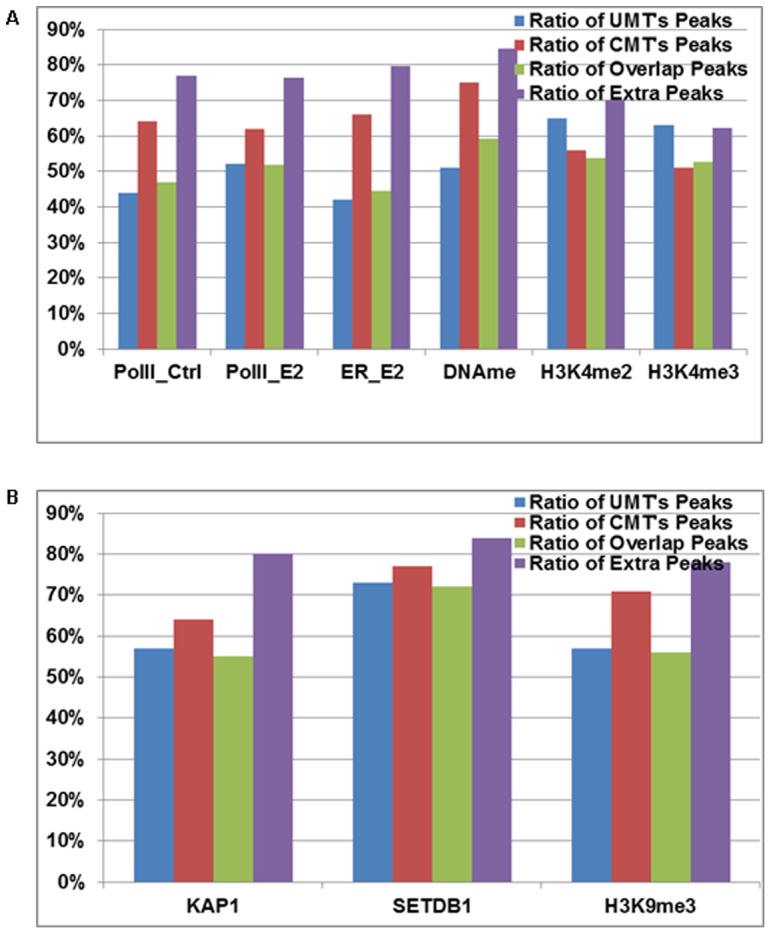
A comparison of the relationship between CMT peaks and repeats regions. **A.** A comparison between ratios of peaks in repeats regions for UMT, CMT, Overlap and Extra peaks using the datasets from MCF7 cells. **B.** A comparison between ratios of peaks in repeats regions for UMT, CMT, Overlap and Extra peaks using K562 datasets.

There are three potentially influential factors for locating a NUT to the reference genome. The first is the distance of a NUT alignment to its closest UMT peak, the second is the peak score of the closest UMT peak of a NUT alignment, and the third is the relationship of the tags with repeats regions, since it is possible that many NUTs are derived from repeats regions. The current version of LONUT only considers the first two factors to derive an empirical formula to locate NUTs. To investigate the possibility of contribution from the third factor, we examined the relationship between the UMT, CMT, and Extra peaks with repeats regions, using the newest version of Repbase [33] as a reference for the repeats regions and found a slight increase in the overall ratio of peaks in repeats regions in the CMT peaks as compared to the UMT peaks, except for two active histone modifications (H3K4me2 and H3K4me3) (**Figure 7A**). We observed that the ratio of Extra peaks in repeats regions is higher than that of Overlap peaks, showing that the addition of NUTs was more valuable in identifying the peaks coming from repeats regions (**Figure 7A,B**). Our results support the concept that the repeats information may influence the assignment of NUTs to genomic regions.

A few recent studies [25]–[26] have demonstrated the importance of assigning NUTs to a unique genomic location and have shown that the tags usually discarded by most researchers are indeed part of functionally relevant regions. For example, Rosenfeld et al. [26] investigated the effectiveness of utilizing NUTs in H3K9me3 datasets and suggested H3K9me3 may have a role in chromatin organization rather than being directly related to gene expression. The study from Chung et al. [26] further illustrated that incorporation of NUTs significantly increased sequencing depth, leading to the detection of new binding sites. However, all of these studies were focused on one or two ChIP-seq datasets. Our study comprehensively investigated the influence of NUTs in many different types of genomic datasets, testing the performance of LONUT on 3 diverse datasets. We examined a comparison of the ratio of Overlap peaks between UMT and CMT peaks, the average of peak scores for CMT, Overlap and Extra peaks, performed de novo motif discovery for the different peaks sets and confirmed the validity of the newly identified peaks using ChIP-PCR. Our results demonstrate the specificity and accuracy of the new alignment tool LONUT.

## Materials and Methods

### Study cases and datasets in this work

We have used three study cases and their associated datasets to demonstrate the performance of our LONUT algorithm and the practical use of the software tool. In Study Case 1, we used ChIP-seq datasets from MCF7 cells, including Pol-II and ERα [25], [34], H3K4me2 and DNA methylation [35], and publically available H3K4me3 (http://genome.ucsc.edu/). In Study Case 2, we used 3 ChIP-seq datasets from K562 cells that detect two different components of a macromolecular complex, KAP1 (a scaffold protein) and SETDB1 (a H3K9me3-specific histone methyltransferase), and the associated modified histone H3K9me3 [31], [32]. Study Case 3 utilized ChIP-seq datasets for three transcription factors, TCF7L2, NRSF and YY1, from four different cell lines; these datasets were from the ENCODE Project (http://genome.ucsc.edu/).

### Establishing an empirical score formula

From a set of many possible alignments on the reference genome for each raw tag produced using the short-tag alignment tool Bowtie aligner [36], we established an empirical score formula and then computed an Empirical Score (ES) for each multiple aligned tag. In the initial alignment, we used default parameters for Bowtie such that each raw tag was allowed to have at most 2 mismatches and at most 100 possible alignments on the reference genome.

To determine an ES, we considered two factors. One was the relative distance (*d*) of each multiple aligned tag to an enriched peak or methylated region, with the assumption that a high quality NUT should be close to or within an enriched region. The other factor considered was the score (*s*) of the enrichment of the binding region identified by our BELT program [29] using the uniquely mapped tags, with the assumption that uniquely mapped tags identify high confidence enriched regions. We then normalized the two measurements to the same numerical scale to equalize the impact of the two factors (see **Results section - Determination of ES formula**). ES was calculated using the following formula, 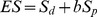
where 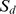 is the normalized distance score using a linear fit with n degree, 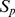
is the peak score of the closest peak, 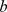 is the normalization factor for the peak score.

We used a polynomial regression model to fit the data points (aligned tags). 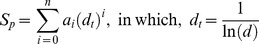
where 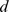 is the relative distance of a NUT alignment to its closest UMT peak, 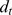 is the transformed distance value, 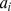 is the coefficient of each term in the polynomial function.

Assume that the total number of points is 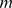, 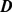 is the designed matrix representing
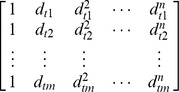
. The least square estimation of the vector of polynomial regression coefficients 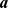 is,
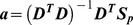
where 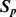 is the vector of peak scores.

### Data preprocessing

LONUT utilizes several existing Bioinformatics tools, such as Bowtie [36] and BELT [29]. Bowtie, an ultrafast, memory-efficient short tag aligner, is used for aligning multiple matched tags to the reference genome. We set the parameters for Bowtie as the default such that each tag is allowed to have at most 2 mismatches and at most 100 copies of alignments on the reference genome for each tags. BELT, a peak-calling program for ChIP-seq data developed in our laboratory, is used for identifying the enriched binding regions from uniquely matched tags.

### Implementation and usage of LONUT

LONUT is implemented in Perl language (Perl 5.88 or above). The source code is platform independent and was tested on a LINUX/UNIX system and HPC cluster system. It also provides a data preprocessing program which requires alignment of non-unique matched tags by Bowtie. The source code is available at http://motif.bmi.ohio-state.edu/LONUT/.

The LONUT tool takes two outputs of results from Bowtie. 1) Users can use the –r option if the input file of LONUT is the –r result of Bowtie, i.e. the input of Bowtie is a sequence file; this is the default option. If the user chooses this option, the name of input file should be in this format: XX_seq_r_bowtie, where XX is the name of input data. 2) Users can use the –q option if the input file of LONUT is the –q result of Bowtie, i.e. the input of Bowtie is a fastq file. If the user chooses this option, the name of input file should be in this format: XX_q_bowtie, where XX is the name of input data.

### ChIP-PCR

To validate newly called peaks from the NUTs, we used ChIP-PCR. The TCF7L2 ChIP assay in HCT116 cells was performed as previously described [37]. Briefly, HCT116 cells (ATCC #CCL-247) were grown in McCoy's 5a Medium supplemented with 10% fetal bovine serum and incubated at 37°C in a humidified 5% CO2 incubator. Cells were harvested at 80% confluence and cross-linked with 1% formaldehyde for 10 min at room temperature. Cross-linking was stopped by the addition of 125 mM glycine for 5 min, and cells were washed twice with ice-cold PBS and scraped from the dish. A nuclear extract was prepared, and chromatin was sonicated to a size of 200–500 bp using the Bioruptor from Diagenode for 45 minutes with cycles of 30 seconds ON, and 90 seconds. The TCF7L2 ChIP assay was performed by incubating 100 µg of HCT116 chromatin with 10 µL of TCF7L2 antibody (Cell Signaling Technology, Danvers, MA, USA; Catalog #C48H11, Lot #2) overnight on a rotating platform at 4 degrees Celsius. Rabbit IgG was used as a negative control (Alpha Diagnostics, Owings Mills, MD, USA; Catalog #210-561-9515). Protein A/G magnetic beads (Pierce, Thermo Scientific, Rockford, IL, USA; Catalog #88803, Lot #NG1561272) were used to collect the immunoprecipitates and eluted ChIP DNA was assayed by PCR using primers in **Figure S1 in File S1**.

## Supporting Information

File S1 **Supplementary figures. Figure S1. The ratio of UMTs/CMTs by averaging every 100 peaks.** A. Pol-II data, B. ER data. It showed the ratio is decreasing along the lower level of ranking peaks, but stable at 0.6 for Pol-II data and 0.35 for ER data, indicating that the newly identified peaks are just as good. **Figure S2. Screenshots for 2 of 274 genes that were identified in the 682 new common peaks are genes that were already in the list of 1,047 genes in the common UMT peaks set.** Top three tracks are UMT peaks for three factors, and lower three are CMT peaks. It shows extra common peaks are from same genes with UMT common peaks. **Figure S3. PCR validation of TCF7L2 peaks in HCT116.** Primers were designed for TCF7L2 peaks visualized using the Integrated Genome Browser (IGB, http://bioviz.org/igb/). Primers for positive sites in the first panel are described in Frietze et al. Genome Biology, 13:R52, 2012. Primers for NUT peaks were designed for peaks identified in the high-threshold CMT dataset (p-value  = 0.99). In order to assay novel peaks as determined by the LONUT algorithm, NUT peaks were chosen as those absent from UMT peak sets. Snapshots were taken for the regions containing NUTS labeled above as ‘1’ and ‘2 as seen in the next figure. All UMT peaks analyzed here were called as peaks in the high-threshold UMT dataset (p-value  = 0.99), but were not present in the CMT dataset of the same threshold. PCR enrichment of these sites demonstrates that these regions are still enriched, even though they are not present in the high-threshold CMT dataset. **Figure S4. Exclusion of UMT peaks from the CMT dataset.** When UMT peaks and NUT peaks are combined to create the CMT dataset, some UMT peaks are excluded from this new dataset as illustrated in the above snapshot from a region on chromosome 1. The peak on the left is a strong UMT peak, was identified at two different thresholds, and was retained in the final CMT dataset. The peak on the right, however was not included in the CMT dataset at the highest threshold (red). The exclusion of this UMT peak from the CMT dataset is a result of of new NUT peaks with larger tag heights being included in the CMT dataset.
(PPTX)

File S2 **Supplementary tables. Table S1.** A comparison of three formulas in a ratio of Overlap and Extra peaks for ChIP-seq data of H3K4me2 in MCF7 cells. **Table S2.** A comparison of three formulas in average peak scores of UMT peaks, CMT peaks, Overlap peaks and Extra peaks for ChIP-seq data of H3K4me2 in MCF7 cells. **Table S3.** A summary of tag distribution of UMTs and NUTs in the first study case. **Table S4.** An overview of UMT and CMT peaks for eight datasets in MCF7 cells. **Table S5.** A summary of Overlap and Extra peaks in eight datasets in the study case 1. **Table S6.** A summary of average UMT peak scores, CMT peak scores, Overlap peak scores, Extra peak scores of eight datasets in the study case 1. **Table S7.** Motif results in the UMT Peaks set of ER_E2 data. **Table S8.** Logos of ER binding motif in the UMT Peaks set of ER_E2 data. **Table S9.** Motif results in the CMT Peaks set of ER_E2 data. **Table S10.** Logos of ER binding motif in the CMT Peaks set of ER_E2 data. **Table S11.** Motif results in the Extra Peaks set of ER_E2 data. **Table S12.** Logos of ER binding motif in the Extra Peaks set of ER_E2 data. **Table S13.** An overview of the tag distribution of UMTs, NUTs and CMTs in KAP1, SETDB1 and H3K9me3 in K562 cell line. **Table S14.** An overview of UMT and CMT peaks in KAP1, SETDB1 and H3K9me3 in K562 cell line. **Table S15.** A summary of Overlap and Extra peaks in KAP1, SETDB1 and H3K9me3 in K562 cell line. **Table S16.** An overview of the tag distribution of UMTs, NUTs and CMTs in NSRF, TCF7L2 and YY1 in four human cell lines. **Table S17.** An overview of UMT and CMT peaks in four datasets of the third study. **Table S18.** A summary of Overlap and Extra peaks in four datasets of the third study case. **Table S19.** A summary of the average UMT's peak scores, CMT's peak scores, Overlap peak scores, Extra peak scores of four datasets in the third study case. **Table S20.** A summary of the comparison of 7,800 UMT peaks to CMT peaks at different thresholds for TCF7L2 in HCT116 cells. **Table S21.** Motif results in UMT's peaks of H1 (NRSF) data. **Table S22.** Weblogos of Motif results in UMT's peaks of H1 (NRSF) data. **Table S23.** Motif results in CMT's peaks of H1 (NRSF) data. **Table 24.** Weblogos of motif results in CMT's peaks of H1 (NRSF) data. **Table 25.** Motif results in UMT's peaks of HCT116 (TCF7L2) data. **Table S26.** Weblogos of motif results in UMT's peaks of HCT116 (TCF7L2) data. **Table S27.** Motif results in CMT's peaks of HCT116 (TCF7L2) data. **Table S28.** Weblogos of motif results in CMT's peaks of HCT116 (TCF7L2) data. **Table S29.** A summary of CR (Chung et al) peaks and CMT peaks both called by BELT.
(DOCX)
