# Supplementary material for: LOcating Non-Unique matched Tags (LONUT) to Improve the Detection of the Enriched Regions for ChIP-seq Data
Source: PLoS One. 2013 Jun 25;8(6):e67788. doi: 10.1371/journal.pone.0067788 (PMC3692479; doi:10.1371/journal.pone.0067788)
Supplement: File S1 — Supplementary figures. Figure S1. The ratio of UMTs/CMTs by averaging every 100 peaks. A. Pol-II data, B. ER data. It showed the ratio is decreasing along the lower level of ranking peaks, but stable at 0.6 for Pol-II data and 0.35 for ER data, indicating that the newly identified peaks are just as good. Figure S2. Screenshots for 2 of 274 genes that were identified in the 682 new common peaks are genes that were already in the list of 1,047 genes in the common UMT peaks set. Top three tracks are UMT peaks for three factors, and lower three are CMT peaks. It shows extra common peaks are from same genes with UMT common peaks. Figure S3. PCR validation of TCF7L2 peaks in HCT116. Primers were designed for TCF7L2 peaks visualized using the Integrated Genome Browser (IGB, http://bioviz.org/igb/). Primers for positive sites in the first panel are described in Frietze et al. Genome Biology, 13:R52, 2012. Primers for NUT peaks were designed for peaks identified in the high-threshold CMT dataset (p-value = 0.99). In order to assay novel peaks as determined by the LONUT algorithm, NUT peaks were chosen as those absent from UMT peak sets. Snapshots were taken for the regions containing NUTS labeled above as ‘1’ and ‘2 as seen in the next figure. All UMT peaks analyzed here were called as peaks in the high-threshold UMT dataset (p-value = 0.99), but were not present in the CMT dataset of the same threshold. PCR enrichment of these sites demonstrates that these regions are still enriched, even though they are not present in the high-threshold CMT dataset. Figure S4. Exclusion of UMT peaks from the CMT dataset. When UMT peaks and NUT peaks are combined to create the CMT dataset, some UMT peaks are excluded from this new dataset as illustrated in the above snapshot from a region on chromosome 1. The peak on the left is a strong UMT peak, was identified at two different thresholds, and was retained in the final CMT dataset. The peak on the right, however was not included in the CMT [file pone.0067788.s001.pptx]

## Slide 1
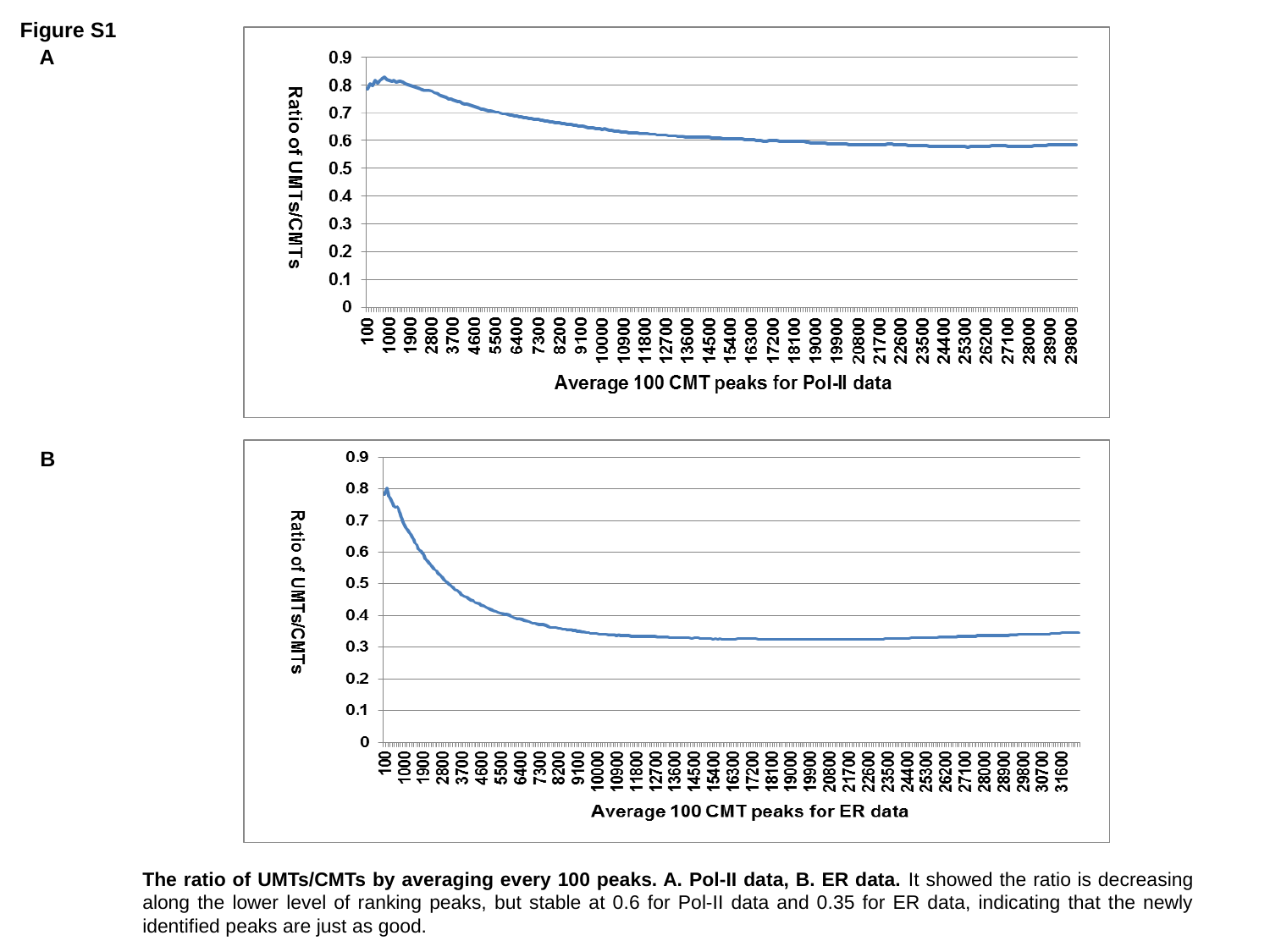

Figure S1
A
B
The ratio of UMTs/CMTs by averaging every 100 peaks. A. Pol-II data, B. ER data. It showed the ratio is decreasing along the lower level of ranking peaks, but stable at 0.6 for Pol-II data and 0.35 for ER data, indicating that the newly identified peaks are just as good.

## Slide 2
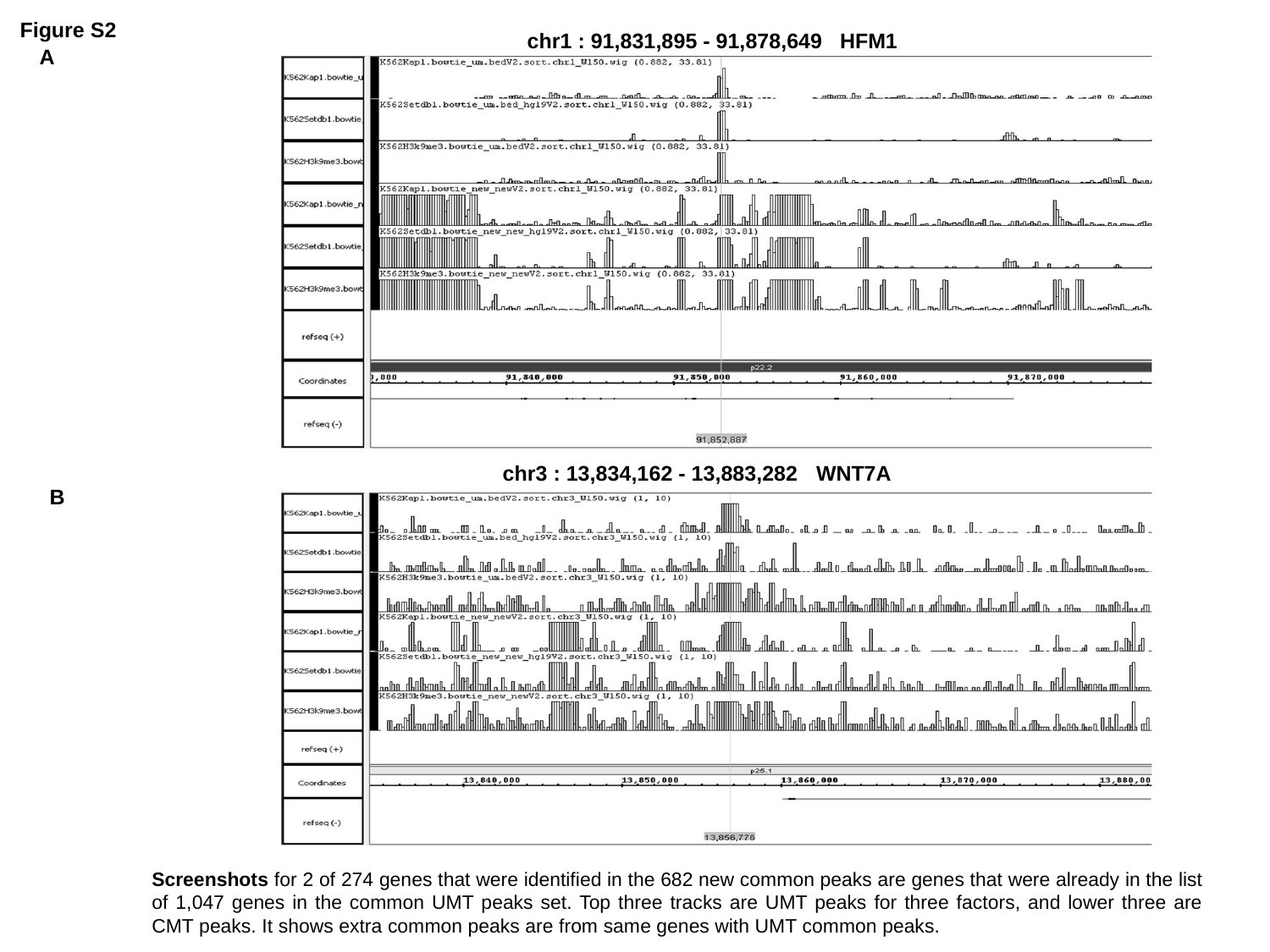

Figure S2
chr1 : 91,831,895 - 91,878,649 HFM1
A
chr3 : 13,834,162 - 13,883,282 WNT7A
B
Screenshots for 2 of 274 genes that were identified in the 682 new common peaks are genes that were already in the list of 1,047 genes in the common UMT peaks set. Top three tracks are UMT peaks for three factors, and lower three are CMT peaks. It shows extra common peaks are from same genes with UMT common peaks.

## Slide 3
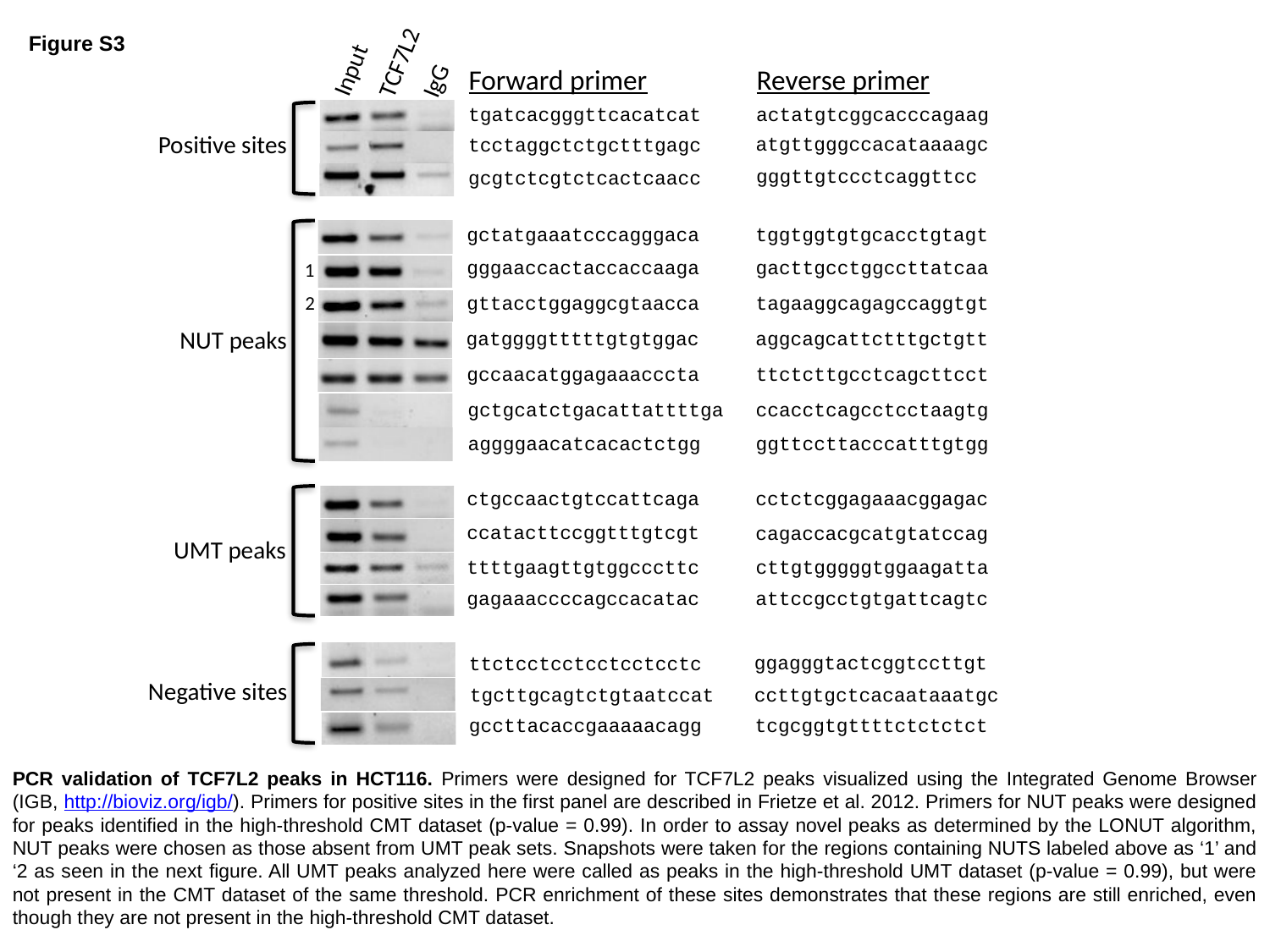

Figure S3
TCF7L2
Input
Forward primer
Reverse primer
IgG
tgatcacgggttcacatcat
actatgtcggcacccagaag
Positive sites
atgttgggccacataaaagc
tcctaggctctgctttgagc
gggttgtccctcaggttcc
gcgtctcgtctcactcaacc
gctatgaaatcccagggaca
tggtggtgtgcacctgtagt
gggaaccactaccaccaaga
gacttgcctggccttatcaa
1
2
gttacctggaggcgtaacca
tagaaggcagagccaggtgt
NUT peaks
aggcagcattctttgctgtt
gatggggtttttgtgtggac
gccaacatggagaaacccta
ttctcttgcctcagcttcct
gctgcatctgacattattttga
ccacctcagcctcctaagtg
aggggaacatcacactctgg
ggttccttacccatttgtgg
ctgccaactgtccattcaga
cctctcggagaaacggagac
ccatacttccggtttgtcgt
cagaccacgcatgtatccag
UMT peaks
ttttgaagttgtggcccttc
cttgtgggggtggaagatta
attccgcctgtgattcagtc
gagaaaccccagccacatac
ggagggtactcggtccttgt
ttctcctcctcctcctcctc
Negative sites
tgcttgcagtctgtaatccat
ccttgtgctcacaataaatgc
gccttacaccgaaaaacagg
tcgcggtgttttctctctct
PCR validation of TCF7L2 peaks in HCT116. Primers were designed for TCF7L2 peaks visualized using the Integrated Genome Browser (IGB, http://bioviz.org/igb/). Primers for positive sites in the first panel are described in Frietze et al. 2012. Primers for NUT peaks were designed for peaks identified in the high-threshold CMT dataset (p-value = 0.99). In order to assay novel peaks as determined by the LONUT algorithm, NUT peaks were chosen as those absent from UMT peak sets. Snapshots were taken for the regions containing NUTS labeled above as ‘1’ and ‘2 as seen in the next figure. All UMT peaks analyzed here were called as peaks in the high-threshold UMT dataset (p-value = 0.99), but were not present in the CMT dataset of the same threshold. PCR enrichment of these sites demonstrates that these regions are still enriched, even though they are not present in the high-threshold CMT dataset.

## Slide 4
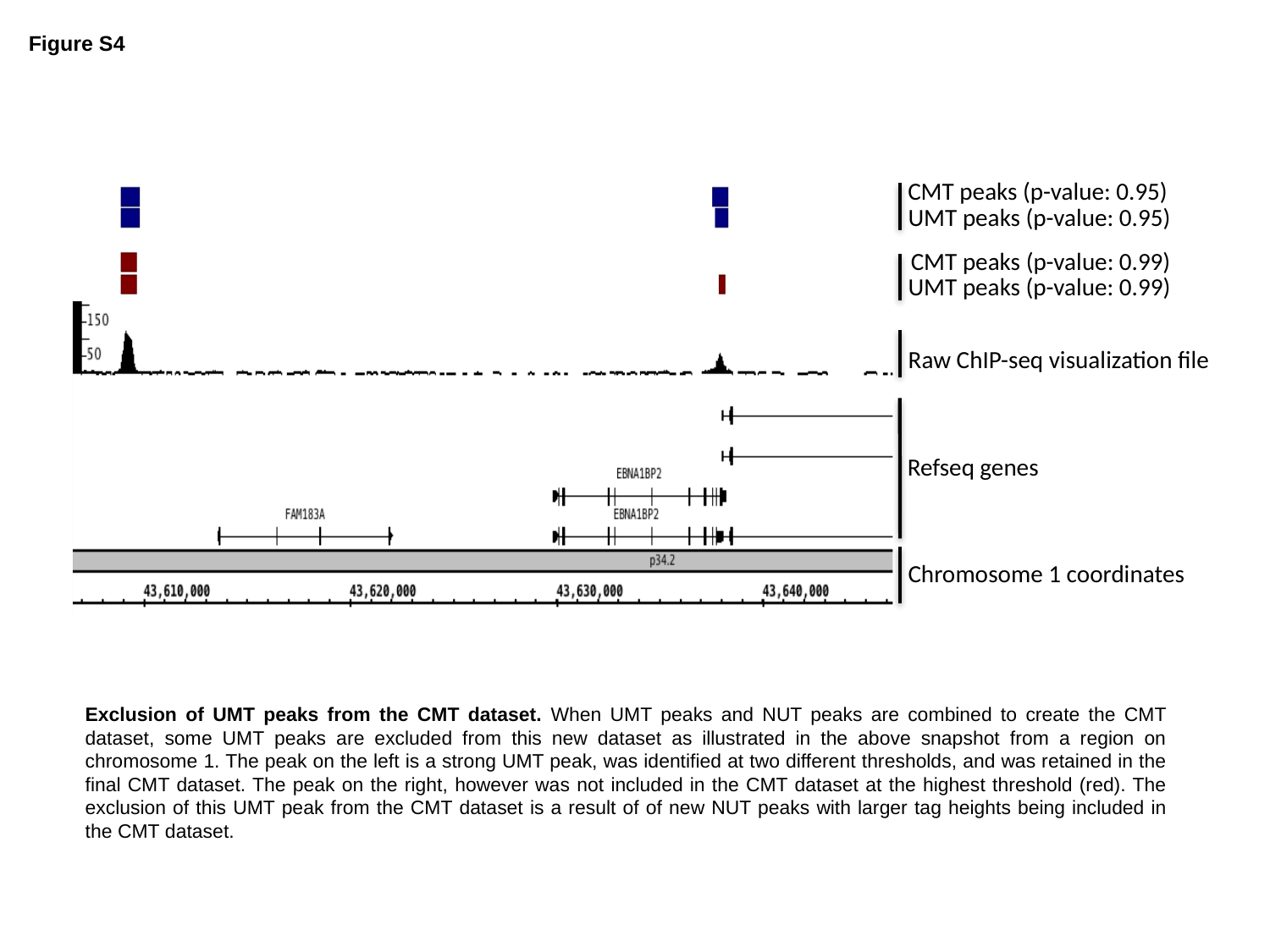

Figure S4
CMT peaks (p-value: 0.95)
UMT peaks (p-value: 0.95)
CMT peaks (p-value: 0.99)
UMT peaks (p-value: 0.99)
Raw ChIP-seq visualization file
Refseq genes
Chromosome 1 coordinates
Exclusion of UMT peaks from the CMT dataset. When UMT peaks and NUT peaks are combined to create the CMT dataset, some UMT peaks are excluded from this new dataset as illustrated in the above snapshot from a region on chromosome 1. The peak on the left is a strong UMT peak, was identified at two different thresholds, and was retained in the final CMT dataset. The peak on the right, however was not included in the CMT dataset at the highest threshold (red). The exclusion of this UMT peak from the CMT dataset is a result of of new NUT peaks with larger tag heights being included in the CMT dataset.
