# Supplementary material for: LOcating Non-Unique matched Tags (LONUT) to Improve the Detection of the Enriched Regions for ChIP-seq Data
Source: PLoS One. 2013 Jun 25;8(6):e67788. doi: 10.1371/journal.pone.0067788 (PMC3692479; doi:10.1371/journal.pone.0067788)
Supplement: File S2 — Supplementary tables. Table S1. A comparison of three formulas in a ratio of Overlap and Extra peaks for ChIP-seq data of H3K4me2 in MCF7 cells. Table S2. A comparison of three formulas in average peak scores of UMT peaks, CMT peaks, Overlap peaks and Extra peaks for ChIP-seq data of H3K4me2 in MCF7 cells. Table S3. A summary of tag distribution of UMTs and NUTs in the first study case. Table S4. An overview of UMT and CMT peaks for eight datasets in MCF7 cells. Table S5. A summary of Overlap and Extra peaks in eight datasets in the study case 1. Table S6. A summary of average UMT peak scores, CMT peak scores, Overlap peak scores, Extra peak scores of eight datasets in the study case 1. Table S7. Motif results in the UMT Peaks set of ER_E2 data. Table S8. Logos of ER binding motif in the UMT Peaks set of ER_E2 data. Table S9. Motif results in the CMT Peaks set of ER_E2 data. Table S10. Logos of ER binding motif in the CMT Peaks set of ER_E2 data. Table S11. Motif results in the Extra Peaks set of ER_E2 data. Table S12. Logos of ER binding motif in the Extra Peaks set of ER_E2 data. Table S13. An overview of the tag distribution of UMTs, NUTs and CMTs in KAP1, SETDB1 and H3K9me3 in K562 cell line. Table S14. An overview of UMT and CMT peaks in KAP1, SETDB1 and H3K9me3 in K562 cell line. Table S15. A summary of Overlap and Extra peaks in KAP1, SETDB1 and H3K9me3 in K562 cell line. Table S16. An overview of the tag distribution of UMTs, NUTs and CMTs in NSRF, TCF7L2 and YY1 in four human cell lines. Table S17. An overview of UMT and CMT peaks in four datasets of the third study. Table S18. A summary of Overlap and Extra peaks in four datasets of the third study case. Table S19. A summary of the average UMT's peak scores, CMT's peak scores, Overlap peak scores, Extra peak scores of four datasets in the third study case. Table S20. A summary of the comparison of 7,800 UMT peaks to CMT peaks at different thresholds for TCF7L2 in HCT116 cells. Table S21. Motif results in U [file pone.0067788.s002.docx]

**Supplementary Notes.**

**The LONUT program.**

**Implementation:**

The main program of LONUT is implemented in Perl language (newer than Perl 5.88). The source code is platform independent and tested on LINUX/UNIX system of cluster system. Its data preprocessing part requires alignment of nucleotide sequences created by Bowtie.

**Input:**

The LONUT program is able to accept several input files, which should contain the classification information of UMTs and NUTs, or the raw sequence information. For the former case, we classify input data based on the raw input data, then merely do the alignment for the NUTs set, meanwhile calling peaks for the UMTs set. For the latter case, we do the alignment for all raw input data, and then classify NUTs and UMTs based on the alignment results.

LONUT could accept the following input format:

Eland, Extended-eland, Fastq, Fasta, Bed, Bowtie alignment result.

**Usage:**

The program could take two kinds of results of Bowtie. Users could use –r option if the input file of LONUT is –r result of Bowtie, i.e. the input of Bowtie is sequence file and this is default. If user uses this option, the name of input file should be in this format: XX_seq_r_bowtie, where XX is the name of input data.

User could use –q option if the input file of LONUT is –q result of Bowtie, i.e. the input of Bowtie is fastq file. If user uses this option, the name of input file should be in this format: XX_q_bowtie, where XX is the name of input data.

**Output**:

The output of LONUT is combined matched tags (CMTs) file that combined a set of newly located tags from NUTs with a set of original UMTs.

**Running time:**

| **Number of raw tags** | **Running time** |
| --- | --- |
| **1 million** | 1 minute |
| **2 million** | 3 minutes |
| **3 million** | 5 minutes 40 seconds |
| **4 million** | 7 minutes 35 seconds |
| **5 million** | 10 minutes |
| **20 million** | 38 minutes |
| **40 million** | 1 hour and 18 minutes |
| **100 million** | 3 hour and 23 minutes |

**Supplementary Tables.**

**Table S1.** A comparison of three formulas in a ratio of Overlap and Extra peaks for ChIP-seq data of H3K4me2 in MCF7 cells.

| **H3K4me2** | **UMT**  **Peak Num.** | **CMT**  **Peak Num.** | **Overlap**  **Peak Num.** | **Overlap**  **Ratio** | **Extra**  **Peak Num.** | **Extra Ratio** |
| --- | --- | --- | --- | --- | --- | --- |
| **ES F[1]** | 27,314 | 31,073 | 27,082 | 87% | 3,991 | 13% |
| **ES F[2]** | 27,314 | 30,099 | 25,979 | 86% | 4,120 | 14% |
| **ES F[2]** | 27,314 | 30,935 | 28,210 | 91% | 2,725 | 9% |

**Table S2.** A comparison of three formulas in average peak scores of UMT peaks, CMT peaks, Overlap peaks and Extra peaks for ChIP-seq data of H3K4me2 in MCF7 cells.

| **H3K4me2** | **Num. of Top**  **peak scores** | **Average**  **UMT Peak Score** | **Average**  **CMT Peak Score** | **Average**  **Overlap Peak Score** | **Average Extra**  **Peak Score** |
| --- | --- | --- | --- | --- | --- |
| **ES F[1]** | 27,314 | 3.739 | 3.803 | 3.844 | 3.479 |
| **ES F[2]** | 27,314 | 3.739 | 3.865 | 3.932 | 3.251 |
| **ES F[3]** | 27,314 | 3.739 | 3.760 | 3.793 | 3.352 |

**Table S3.** A summary of tag distribution of UMTs and NUTs in the first study case.

|  | **Total Tags** | **UMT** | **UMT Ratio** | **NUT** | **NUT Ratio** |
| --- | --- | --- | --- | --- | --- |
| **PolII_Ctrl** | 4,872,460 | 3,765,201 | 77% | 1,107,259 | 23% |
| **PolII_E2** | 10,810,080 | 7,968,585 | 74% | 2,841,495 | 26% |
| **ER_E2** | 12,652,745 | 8,092,251 | 64% | 4,560,494 | 36% |
| **DNAme** | 37,485,118 | 24,876,183 | 66% | 12,608,935 | 34% |
| **H3K4me2** | 2,910,475 | 2,417,878 | 83% | 492,597 | 17% |
| **H3K4me3** | 34,800,428 | 28,152,818 | 81% | 6,647,610 | 19% |

Table S4. An overview of UMT and CMT peaks for eight datasets in MCF7 cells.

|  | **Bin Size  (w)** | **Threshold  (P)** | **UMT Peak Num.  (FDR)** | **Bin Size  (w)** | **Threshold  (P)** | **CMT Peak Num.  (FDR)** |
| --- | --- | --- | --- | --- | --- | --- |
| **PolII_Ctrl** | 160 | 0.94 | 18,988  (8.19%) | 160 | 0.94 | 26,537  (6.25%) |
| **PolII_E2** | 150 | 0.95 | 22,319  (2.14%) | 150 | 0.94 | 30,083  (2.55%) |
| **ER_E2** | 150 | 0.95 | 23,270  (2.55%) | 150 | 0.96 | 32,485  (0.99%) |
| **DNAme** | 150 | 0.97 | 15,156  (1.89%) | 150 | 0.96 | 24,775  (1.14%) |
| **H3K4me2** | 200 | 0.93 | 24,467  (3.35%) | 150 | 0.93 | 31,073  (2.64%) |
| **H3K4me3** | 150 | 0.96 | 16,745  (1.41%) | 150 | 0.94 | 22,736  (4.26%) |

**Table S5.** A summary of Overlap and Extra peaks in eight datasets in the study case 1.

|  | **UMT Peak**  **Num.** | **CMT Peak Num.** | **Overlap Peak**  **Num** | **Overlap**  **Ratio** | **Extra Peak**  **Num.** | **Extra**  **Ratio** |
| --- | --- | --- | --- | --- | --- | --- |
| **PolII_Ctrl** | 18,988 | 26,537 | 11,726 | 44% | 14,811 | 56% |
| **PolII_E2** | 22,319 | 30,083 | 17,835 | 59% | 12,248 | 41% |
| **ER_E2** | 23,270 | 32,485 | 13,518 | 42% | 18,967 | 58% |
| **DNAme** | 15,156 | 24,775 | 8,592 | 35% | 16,183 | 65% |
| **H3K4me2** | 24,467 | 31,073 | 23,839 | 77% | 7,234 | 23% |
| **H3K4me3** | 16,745 | 22,736 | 16,745 | 74% | 5,991 | 26% |

**Table S6.** A summary of average UMT peak scores, CMT peak scores, Overlap peak scores, Extra peak scores of eight datasets in the study case 1.

|  | **Num. of Peaks** | **Average**  **UMT**  **Peak Score** | **Average**  **CMT**  **Peak Score** | **Average**  **Overlap**  **Peak Score** | **Average Extra**  **Peak Score Score Score** |
| --- | --- | --- | --- | --- | --- |
| **PolII_Ctrl** | 18,988 | 2.605 | 3.396 | 3.447 | 3.360 |
| **PolII_E2** | 22,319 | 4.412 | 4.902 | 5.024 | 4.728 |
| **ER_E2** | 23,270 | 3.876 | 4.628 | 4.822 | 4.501 |
| **DNAme** | 15,156 | 5.323 | 6.181 | 6.106 | 6.219 |
| **H3K4me2** | 24,467 | 3.739 | 3.803 | 3.844 | 3.479 |
| **H3K4me3** | 16,745 | 7.029 | 7.251 | 7.336 | 6.755 |

**Table S7.** Motif results in the UMT Peaks set of ER_E2 data.

| **Known**  **Motif** | **Located Motif** | **E-Value** | **Alignment** |
| --- | --- | --- | --- |
| **ER_Q6** | ER_E2_UMTW3 | 5.08E-04 | ******CAGGGTGACC***  NNNGGTCANNNNNNNYNNN |
| **ER_Q6** | ER_E2_UMTW4 | 9.65E-04 | ***GGTCAC**********  NNNGGTCANNNNNNNYNNN |
| **ER_Q6** | ER_E2_UMTW5 | 7.04E-04 | *CAGGTCAS**********  NNNRNNNNNNNTGACCNNN |
| **ER_Q6** | ER_E2_UMTM1 | 3.04E-03 | NNRGGNCANNSTGACCTN*  NNNGGTCANNNNNNNYNNN |
| **ER_Q6** | ER_E2_UMTM11 | 2.82E-03 | NNRGGKCANKSTGACCTNNN  NNNGGTCANNNNNNNYNNN |

**Table S8.** Logos of ER binding motif in the UMT Peaks set of ER_E2 data.

| **Motif** | **Motif Logo** |
| --- | --- |
| **ER_Q6** |  |
| **ER_E2_UMTW3** |  |
| **ER_E2_UMTW4** |  |
| **ER_E2_UMTW5** |  |
| **ER_E2_UMT1** |  |
| **ER_E2_UMTM11** |  |

**Table S9.** Motif results in the CMT Peaks set of ER_E2 data.

| **Known**  **Motif** | **Located Motif** | **E-Value** | **Alignment** |
| --- | --- | --- | --- |
| **ER_Q6_02** | ER_E2_CMTW1 | 1.35E-04 | ****TGACCT*  RNNNTGACCTN |
| **ER_Q6** | ER_E2_CMTW2 | 7.85E-04 | *CAGGTCAS**********  NNNRNNNNNNNTGACCNNN |
| **ER_Q6** | ER_E2_CMTW3 | 5.78E-04 | ***GGTCACCCTG******  NNNRNNNNNNNTGACCNNN |
| **ER_Q6** | ER_E2_CMTW4 | 1.01E-03 | ***GGTCAC**********  NNNGGTCANNNNNNNYNNN |
| **ER_Q6** | ER_E2_CMTF1 | 6.95E-04 | ******CANNSTGACCTN*  NNNGGTCANNNNNNNYNNN |
| **ER_Q6** | ER_E2_CMTM1 | 2.03E-03 | *NAGGKCANNNTGACCTNN  NNNGGTCANNNNNNNYNNN |
| **ER_Q6** | ER_E2_CMTM11 | 2.03E-03 | NNRGGKCANKGTGACCTNNN  NNNGGTCANNNNNNNYNNN |

**Table S10.** Logos of ER binding motif in the CMT Peaks set of ER_E2 data.

| **Motif** | **Motif Logo** |
| --- | --- |
| ER_Q6_02 |  |
| ER_Q6 |  |
| ER_E2_CMTW1 |  |
| ER_E2_CMTW2 |  |
| ER_E2_CMTW3 |  |
| ER_E2_CMTW4 |  |
| ER_E2_CMTF1 |  |
| ER_E2_CMTM1 |  |
| ER_E2_CMTM11 |  |

**Table S11.** Motif results in the Extra Peaks set of ER_E2 data.

| **Known**  **Motif** | **Located Motif** | **E-Value** | **Alignment** |
| --- | --- | --- | --- |
| **ER_Q6_02** | ER_E2extra_top1000W1 | 1.35E-04 | *AGGTCA****  NAGGTCANNNY |
| **ER_Q6** | ER_E2extra_top1000W2 | 1.31E-04 | **AGGTCACC*********  NNNRNNNNNNNTGACCNNN |
| **ER_Q6** | ER_E2extra_top1000W3 | 1.0E-03 | ***GGTCACCCTG******  NNNRNNNNNNNTGACCNNN |
| **ER_Q6** | ER_E2extra_top1000W4 | 9.82E-04 | **********GTGACC***  NNNGGTCANNNNNNNYNNN |
| **ER_Q6** | ER_E2extra_top1000F1 | 6.45E-04 | ******CANNNTGACCTN*  NNNGGTCANNNNNNNYNNN |
| **ER_Q6** | ER_E2extra_top1000M1 | 1.52E-03 | **RGGKCANGNTGACCYNNN  NNNGGTCANNNNNNNYNNN* |
| **ER_Q6_02** | ER_E2extra_mid1000W1 | 1.36E-04 | ****TGACCT*  RNNNTGACCTN |
| **ER_Q6** | ER_E2extra_mid1000W2 | 1.38E-04 | **AGGTCACC*********  NNNRNNNNNNNTGACCNNN |
| **ER_Q6** | ER_E2extra_mid1000W3 | 5.09E-04 | ******CAGGGTGACC***  NNNGGTCANNNNNNNYNNN |
| **ER_Q6** | ER_E2extra_mid1000W4 | 9.09E-04 | ***GGTCAC**********  NNNGGTCANNNNNNNYNNN |
| **ER_Q6_02** | ER_E2extra_low1000W1 | 1.36E-04 | ****TGACCT*  RNNNTGACCTN |
| **ER_Q6** | ER_E2extra_low1000W3 | 8.92e-04 | ***GGTCAC**********  NNNGGTCANNNNNNNYNNN |

**Table S12.** Logos of ER binding motif in the Extra Peaks set of ER_E2 data.

| **Motif** | **Motif Logo** | **Motif** | **Motif Logo** |
| --- | --- | --- | --- |
| ER_Q6_02 | 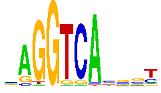 | ER_E2extra_top1000M1 | 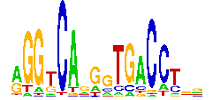 |
| ER_Q6 | 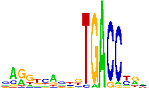 | ER_E2extra_mid1000W1 | 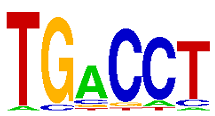 |
| ER_E2extra_top1000W1 | 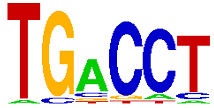 | ER_E2extra_mid1000W2 | 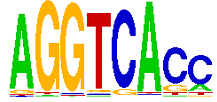 |
| ER_E2extra_top1000W2 | 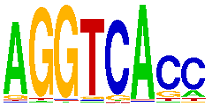 | ER_E2extra_mid1000W3 | 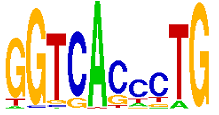 |
| ER_E2extra_top1000W3 | 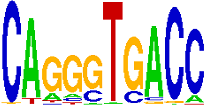 | ER_E2extra_mid1000W4 | 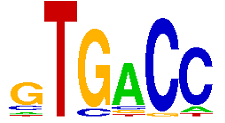 |
| ER_E2extra_top1000W4 | 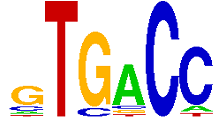 | ER_E2extra_low1000W1 | 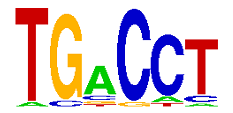 |
| ER_E2extra_top1000F1 | 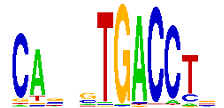 | ER_E2extra_low1000W3 | 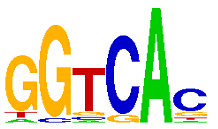 |

**Table S13.** An overview of the tag distribution of UMTs, NUTs and CMTs in KAP1, SETDB1 and H3K9me3 in K562 cell line.

| **Factors** | **Total Tags** | **UMT** | **UMT Ratio** | **NUT** | **NUT Ratio** | **CMT** |
| --- | --- | --- | --- | --- | --- | --- |
| **KAP1** | 35,171,595 | 25,909,139 | 74% | 9,262,456 | 26% | 35,171,595 |
| **SETDB1** | 23,818,887 | 15,722,857 | 66% | 8,096,030 | 34% | 23,818,887 |
| **H3K9me3** | 59,540,653 | 41,303,854 | 69% | 18,236,799 | 31% | 59,540,653 |

**Table S14**. An overview of UMT and CMT peaks in KAP1, SETDB1 and H3K9me3 in K562 cell line.

|  | **Bin size  (w)** | **Threshold  (P)** | **UMT’s peak Num. (FDR)** | **Bin size  (w)** | **Threshold  (P)** | **CMT’s peak Num. (FDR)** |
| --- | --- | --- | --- | --- | --- | --- |
| **KAP1** | 150 | 0.98 | 8,545 (6.64%) | 150 | 0.98 | 11,701 (1.65%) |
| **SETDB1** | 150 | 0.95 | 20,705 (7.25%) | 150 | 0.94 | 35,073  (1.92%) |
| **H3K9me3** | 150 | 0.95 | 22,916 (6.37%) | 150 | 0.93 | 36,532 (4.21%) |

**Table S15.** A summary of Overlap and Extra peaks in KAP1, SETDB1 and H3K9me3 in K562 cell line.

| **Factors** | **UMT’s Peak  Num.** | **CMT’s Peak  Num.** | **Overlap Peak Num.   (Ratio)** | **Extra Peak Num.  (Ratio)** |
| --- | --- | --- | --- | --- |
| **KAP1** | 8,545 | 11,701 | 6,318 (54%) | 5,383 (46%) |
| **SETDB1** | 20,705 | 35,073 | 18, 051 (51%) | 17,022 (49%) |
| **H3K9me3** | 22,916 | 36,532 | 18,894 (52%) | 17,638 (48%) |

**Table S16.** An overview of the tag distribution of UMTs, NUTs and CMTs in NSRF, TCF7L2 and YY1 in four human cell lines.

| **Cell(TF)** | **Total Tags** | **UMT** | **UMT Ratio** | **NUT** | **NUT Ratio** | **CMT** |
| --- | --- | --- | --- | --- | --- | --- |
| **GM12878 (NRSF)** | 31,008,376 | 18,221,832 | 59% | 12,786,544 | 41% | 31,008,376 |
| **H1 (NRSF)** | 38,340,465 | 29,312,501 | 76% | 9,027,964 | 24% | 38,340,465 |
| **HCT116 (TCF7L2)** | 17,992,833 | 13,045,108 | 73% | 4,947,725 | 27% | 17,992,833 |
| **K562 (YY1)** | 19,457,626 | 12,827,352 | 66% | 6,630,274 | 34% | 19,457,626 |

**Table S17**. An overview of UMT and CMT peaks in four datasets of the third study.

|  | **Bin size  (w)** | **Threshold  (P)** | **UMT’s peak Num. (FDR)** | **Bin size  (w)** | **Threshold  (P)** | **CMT’s peak Num. (FDR)** |
| --- | --- | --- | --- | --- | --- | --- |
| **GM12878 (NRSF)** | 150 | 0.95 | 27,479 (3.15%) | 150 | 0.95 | 32,516 (1.15%) |
| **H1 (NRSF)** | 150 | 0.95 | 31,160 (1.32%) | 150 | 0.95 | 34,555 (1.18%) |
| **HCT116 (TCF7L2)** | 150 | 0.95 | 32,355 (0.59%) | 150 | 0.95 | 34,906 (0.33%) |
| **K562 (YY1)** | 150 | 0.95 | 17,143 (6.21%) | 150 | 0.95 | 32,119 (1.05%) |

**Table S18.** A summary of Overlap and Extra peaks in four datasets of the third study case.

| **Cell(TF)** | **UMT’s Peak  Num.** | **CMT’s Peak  Num.** | **Overlap Peak Num. (Ratio)** | **Extra Peak Num. (Ratio)** |
| --- | --- | --- | --- | --- |
| **GM12878 (NRSF)** | 27,479 | 32,516 | 14,602 (45%) | 17,914 (55%) |
| **H1 (NRSF)** | 31,160 | 34,555 | 14,513 (42%) | 20,042 (58%) |
| **HCT116 (TCF7L2)** | 32,355 | 34,906 | 21,642 (62%) | 13,264(38%) |
| **K562 (YY1)** | 17,143 | 32,119 | 19,593 (61%) | 12,526 (39%) |

Table S19. A summary of the average UMT’s peak scores, CMT’s peak scores, Overlap peak scores, Extra peak scores of four datasets in the third study case.

| **Cell (TF)** | **Num. of Top  peak scores involved** | **Average UMT’s  Peak Score** | **Average CMT’s  Peak Score** | **Average Overlap Peak Score** | **Average Extra Peak Score** | **Average Overlap Peak Score/ Average Extra Peak Score** |
| --- | --- | --- | --- | --- | --- | --- |
| **GM12878 (NRSF)** | 27,479 | 4.252 | 5.898 | 6.386 | 5.913 | 1.080 |
| **H1 (NRSF)** | 31,160 | 4.615 | 5.541 | 6.012 | 5.452 | 1.103 |
| **HCT116 (TCF7L2)** | 32,355 | 4.769 | 5.348 | 5.526 | 5.452 | 1.013 |
| **K562 (YY1)** | 17,143 | 3.850 | 5.501 | 5.916 | 5.585 | 1.059 |

**Table S20**. A summary of the comparison of 7,800 UMT peaks to CMT peaks at different thresholds for TCF7L2 in HCT116 cells.

| **UMT Parameters**  **(Peaks Num.)** | **CMT Parameters**  **(Peaks Num.)** | **Overlap Peaks Num.**  **(%)** |
| --- | --- | --- |
| 0.99/150  (7,800) | 0.95/150  (34,906 ) | 7,800 (100%) |
| 0.99/150  (7,800) | 0.96/150  (28,620 ) | 7,800 (100%) |
| 0.99/150  (7,800) | 0.97/150  (22,198) | 7,800 (100%) |
| 0.99/150  (7,800) | 0.98/150  (15,044) | 7,800 (100%) |
| 0.99/150  (7,800) | 0.99/150  (7,706) | 5,389 (69%) |

Table S21. Motif results in UMT’s peaks of H1 (NRSF) data.

| **Known Motif** | **Located Motif** | **E-Value** | **Alignment** |
| --- | --- | --- | --- |
| **NRSF_01** | H1hescNrsfPcr1x_umFactorW1 | 7.21E-05 | ************GGTGCT*** GSYGCTGTCCGTGGTGCTGAA |
| **NRSF_01** | H1hescNrsfPcr1x_umFactorF1 | 4.59E-03 | ************GGTGCTGANNNN GSYGCTGTCCGTGGTGCTGAA*** |
| **NRSF_01** | H1hescNrsfPcr1x_umFactorM1 | 9.68E-09 | *TCAGCACCNNGGACAGCN** TTCAGCACCACGGACAGCRSC |
| **NRSF_01** | H1hescNrsfPcr1x_umFactorM11 | 2.54E-14 | NGGNGCTGTCCNNGGTGCTGAN *GSYGCTGTCCGTGGTGCTGAA |

Table S22. Weblogos of Motif results in UMT’s peaks of H1 (NRSF) data.

| **Motif** | **Weblogo** |
| --- | --- |
| **NRSF_01** |  |
| **H1hescNrsfPcr1x_umFactorW1** |  |
| **H1hescNrsfPcr1x_umFactorF1** |  |
| **H1hescNrsfPcr1x_umFactorM11** |  |

Table S23. Motif results in CMT’s peaks of H1 (NRSF) data.

| **Known Motif** | **Located Motif** | **E-Value** | **Alignment** |
| --- | --- | --- | --- |
| **NRSF_01** | H1hescNrsfPcr1x_combinedFactorW1 | 7.09E-05 | ************GGTGCT***  GSYGCTGTCCGTGGTGCTGAA |
| **NRSF_01** | H1hescNrsfPcr1x_combinedFactorM1 | 6.59E-09 | **NGCTGTCCNNGGTGCTGA*  GSYGCTGTCCGTGGTGCTGAA |
| **NRSF_01** | H1hescNrsfPcr1x_combinedFactorM11 | 0.00E-0 | *TCAGCACCNYGGACAGCNCC  TTCAGCACCACGGACAGCRSC |

Table 24. Weblogos of motif results in CMT’s peaks of H1 (NRSF) data.

| **Motif** | **Weblogo** |
| --- | --- |
| **NRSF_01** |  |
| **H1hescNrsfPcr1x_combinedFactorW1** |  |
| **H1hescNrsfPcr1x_combinedFactorM1** |  |
| **H1hescNrsfPcr1x_combinedFactorM11** |  |

Table 25. Motif results in UMT’s peaks of HCT116 (TCF7L2) data

| **Known Motif** | **Located Motif** | **E-Value** | **Alignment** |
| --- | --- | --- | --- |
| **TCF7L2_Q5** | Hct116Tcf7l2_umFactorF1 | 5.73E-03 | WTCAAAGNNNNN WTCAAAGS**** |
| **TCF7L2_Q5** | Hct116Tcf7l2_umFactorM13 | 1.65E-03 | NNNNNNNNNNASATCAAAGN ************WTCAAAGS |
| **TCF7L2_Q5** | Hct116Tcf7l2_umFactorM2 | 1.67E-03 | NNNNNASATCAAAGNN *******WTCAAAGS* |

Table S26. Weblogos of motif results in UMT’s peaks of HCT116 (TCF7L2) data

| **Motif** | **Weblogo** |
| --- | --- |
| **TCF7L2_Q5** |  |
| **Hct116Tcf7l2_umFactorF1** |  |
| **Hct116Tcf7l2_umFactorM13** |  |
| **Hct116Tcf7l2_umFactorM2** |  |

Table S27. Motif results in CMT’s peaks of HCT116 (TCF7L2) data

| **Known Motif** | **Located Motif** | **E-Value** | **Alignment** |
| --- | --- | --- | --- |
| **TCF7L2_Q5** | Hct116Tcf7l2_combinedFactorW1 | 4.59  E-05 | *TCAAAG*  WTCAAAGS |
| **TCF7L2_Q5** | Hct116Tcf7l2_combinedFactorF1 | 5.88  E-04 | NNNNNWTCAAAG* *****WTCAAAGS |
| **TCF7L2_Q5** | Hct116Tcf7l2_combinedFactorM12 | 2.19  E-03 | NNNNNNNNNASATCAAAGNN  ***********WTCAAAGS* |
| **TCF7L2_Q5** | Hct116Tcf7l2_combinedFactorM13 | 4.01  E-03 | ACATTCAAAAGCTAGCAGAAGGCAA  ***WTCAAAGS************** |

Table S28. Weblogos of motif results in CMT’s peaks of HCT116 (TCF7L2) data.

| **Motif** | **Weblogo** |
| --- | --- |
| **TCF7L2_Q5** |  |
| **Hct116Tcf7l2_combinedFactorW1** |  |
| **Hct116Tcf7l2_combinedFactorF1** |  |
| **Hct116Tcf7l2_combinedFactorM12** |  |
| **Hct116Tcf7l2_combinedFactorM13** |  |

**Table S29.** A summary of CR (Chung et al) peaks and CMT peaks both called by BELT.

| **Thresholds** | **CR Peaks Num. (FDR)** | **CMT Peaks Num. (FDR)** | **Overlap Num. (%)** |
| --- | --- | --- | --- |
| 0.95 | 33,750 (1.84%) | 34,906 (0.53%) | 22,001 (63%) |
| 0.96 | 27,783 (0.66%) | 28,620 (0.77%) | 18,104 (63%) |
| 0.97 | 21,427 (0.44%) | 22,198 (0.35%) | 14,206 (64%) |
| 0.98 | 15,199 (0.17%) | 15,044 (0.21%) | 10,308 (69%) |
| 0.99 | 8,022 (0.013%) | 7,706 (0.02%) | 6,298 (82%) |
